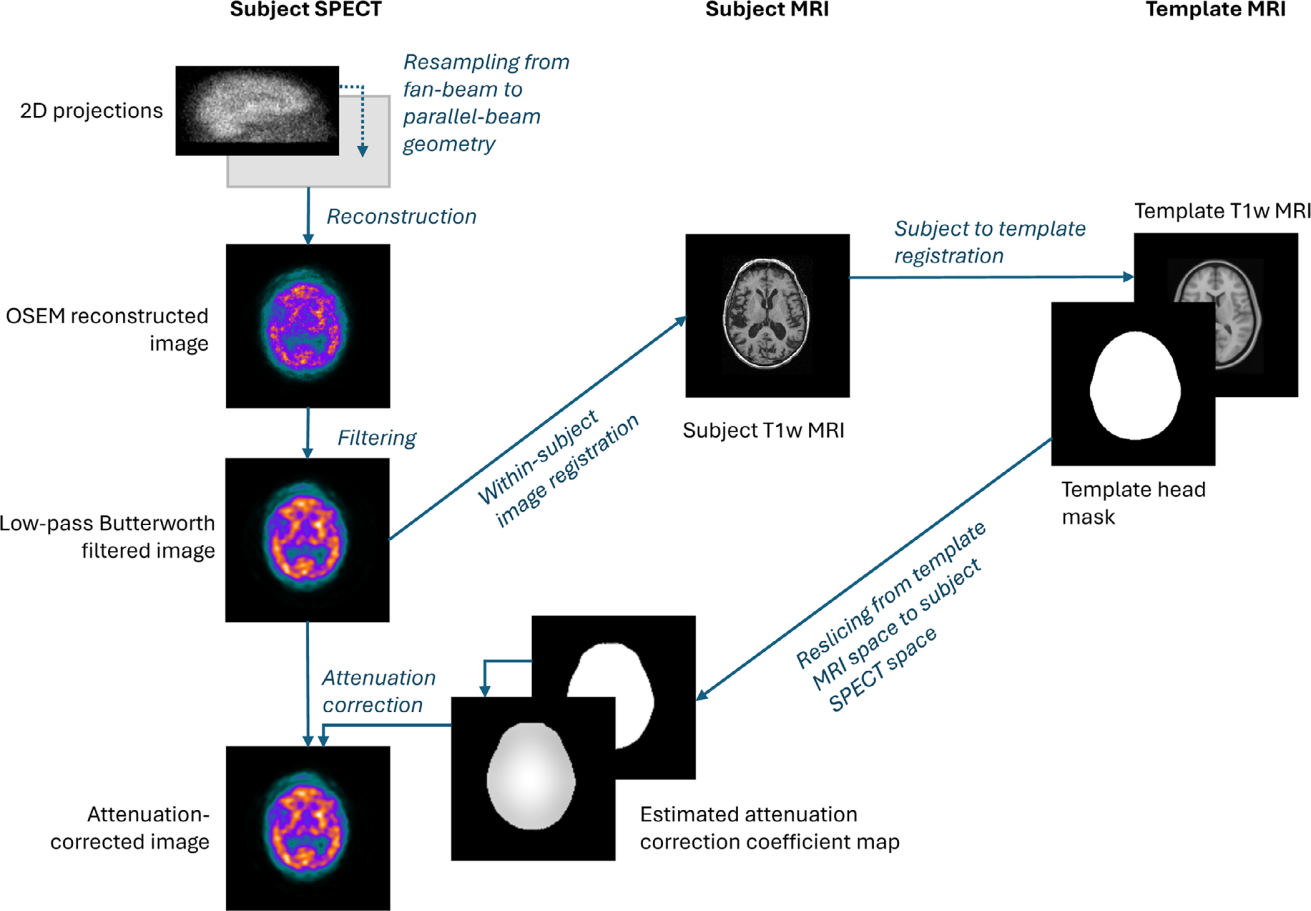# Automating SPECT Reconstruction for Dementia Research Initiatives

**DOI:** 10.1002/alz70856_107469

**Published:** 2026-01-09

**Authors:** Suraj Sudheendra, Gregory Szilagyi, Christopher JM Scott, Vincent Gaudet, Sandra E. Black, Katherine Zukotynski

**Affiliations:** ^1^ Sunnybrook Research Institute, Toronto, ON, Canada; ^2^ Dr. Sandra E. Black Centre for Brain Resilience and Recovery, LC Campbell Cognitive Neurology, Hurvitz Brain Sciences Program, Sunnybrook Research Institute, University of Toronto, Toronto, ON, Canada; ^3^ Department of Electrical and Computer Engineering, University of Waterloo, Waterloo, ON, Canada; ^4^ Sunnybrook Research Institute, University of Toronto, Toronto, ON, Canada

## Abstract

**Background:**

Dementia research initiatives are important for advancing our understanding of neurodegenerative diseases. While there is much discussion regarding Positron Emission Tomography (PET) radiopharmaceuticals for detecting amyloid and tau deposition in the brain, understanding cerebral perfusion is also key. Single Photon Emission Computed Tomography (SPECT) can show abnormalities in cerebral blood flow within key brain regions such as the basal ganglia and medial temporal lobe, offering a potential biomarker for personalized therapeutic strategies in patients with cognitive decline. The dementia research initiative at the Sunnybrook Research Institute has acquired multiple SPECT scans in numerous participants, necessitating the creation of automated image processing pipelines for data analysis.

**Method:**

An automated pipeline is being developed for processing brain perfusion SPECT. Using 5 datasets, projection data were resampled from fan‐beam to parallel‐beam geometry and reconstructed with the Ordered Subset Expectation Maximization (OSEM) algorithm. Noise reduction was then applied using a low‐pass Butterworth filter to attenuate high‐frequency signals in the reconstructed volume. Within‐subject inter‐modal image registration was used to obtain coefficient estimates for attenuation correction from an MRI template head mask resliced into subject SPECT space. Anatomical regions of interest in subject MRI space were used for the regional analysis of relative cerebral blood flow. Pairwise Structural Similarity Index Measure (SSIM) between reconstructions was used to survey the degree of influence of OSEM and filter parameter values on the perceived visual quality of reconstructed images.

**Result:**

Our automated pipeline for analyzing SPECT data has been implemented with noise filtering to enhance image quality, attenuation correction, and image registration to enable comparison with MRI (as illustrated in Figure 1). The pipeline will be used with our existing dataset of several thousand brain perfusion SPECT images, such that comparison of clinical cognitive function with cerebral perfusion can be assessed.

**Conclusion:**

This study focuses on the considerations needed to implement an automated pipeline for processing brain perfusion SPECT data in patients with cognitive issues. It illustrates a possible approach to the analysis of large brain perfusion SPECT datasets helping to support the incorporation of perfusion data in personalized dementia therapy programs.